# The Impact of the Accelerometer Sampling Rate on the Performance of Machine and Deep Learning Models in Wearable Fall-Detection Systems

**DOI:** 10.3390/s26010162

**Published:** 2025-12-26

**Authors:** Manny Villa, Eduardo Casilari

**Affiliations:** 1 Programa de Ingeniería Electrónica, Universidad de Investigación y Desarrollo (UDI), Bucaramanga 680001, Colombia; 2 Departamento de Tecnología Electrónica, Instituto TELMA, Universidad de Málaga, 29071 Málaga, Spain; ecasilari@uma.es

**Keywords:** fall detection systems, wearable devices, sampling frequency, deep learning, CNN-LSTM, inertial sensors, IoT, energy efficiency, telemonitoring

## Abstract

Population aging has intensified the prevalence of falls among older adults, making automatic Fall Detection Systems (FDS) a key component of telemonitoring and remote care. Among wearable-based approaches, inertial sensors, particularly accelerometers, offer an effective and low-cost alternative for continuous monitoring. However, the impact of the selection of the sampling frequency on model performance remains insufficiently explored. This work seeks to determine the sampling rate that best balances accuracy, stability, and computational efficiency in wearable FDS. Five representative algorithms (CNN-LSTM, CNN, LSTM-BN, k-NN, and SVM) were trained and evaluated using the SisFall dataset at 10, 20, 50, and 100 Hz, followed by a multi-stage validation including the real-fall repositories FARSEEING and Free From Falls, as well as a seven-day continuous monitoring test under real-life conditions. The results show that deep learning architectures consistently outperform traditional classifiers, with the CNN-LSTM model at 20 Hz achieving the best balance of accuracy (98.9%), sensitivity (96.7%), and specificity (99.6%), while maintaining stable performance across all validations. The observed consistency indicates that intermediate frequencies, around 20 Hz and down to 10 Hz, provide sufficient temporal resolution to capture fall dynamics while reducing data volume, which translates into more efficient energy usage compared to higher sampling rates. Overall, these findings establish a solid empirical foundation for designing next-generation wearable fall-detection systems that are more autonomous, robust, and sustainable in long-term IoT-based monitoring environments.

## 1. Introduction

The aging of the global population has become one of the major public health challenges of the twenty-first century. According to projections by the World Health Organization (WHO), by 2050 the number of people aged 60 years and older is expected to surpass 2.1 billion, accounting for about 22% of the world’s population [1]. This demographic shift has significantly increased the occurrence of falls among the elderly, placing them among the main causes of disease burden and death within this age cohort. An estimated 684,000 individuals annually die from fall-related injuries, the majority of whom are over 60 years old [2,3].

Falls represent not only a traumatic physical incident but also a major cause of secondary injuries such as hip fractures, traumatic brain damage, and other musculoskeletal damage. Beyond the physical impact, falls commonly lead to psychological and social repercussions -such as loss of confidence, fear of recurrence, reduced mobility, and even depression. These factors collectively contribute to a gradual decline in quality of life and personal autonomy [4,5]. Previous studies have reported that between 28% and 35% of adults aged 65 years or older experience at least one fall per year, a figure that increases to 42% among individuals over 70 [4]. Nearly half of those affected are unable to stand up without assistance. Consequently, after a fall, a significant proportion remain on the ground for prolonged periods, thereby raising the likelihood of severe medical complications [6].

In this regard, automatic Fall Detection Systems (FDS) have emerged as appealing tools for remote health monitoring and assistance of older individuals or those with limited mobility. These technologies provide instant notifications to caregivers or medical staff, allowing rapid responses that help prevent severe outcomes [7]. Among the different technological strategies, three primary approaches are typically distinguished: vision-based methods, ambient sensor networks, and wearable systems [8]. The first two, often referred to as context-aware systems, face major challenges such as privacy issues, reliance on indoor settings, and high deployment costs. Conversely, wearable devices stand out for their affordability, portability, continuous monitoring capability, and energy efficiency [9]. As the main disadvantage, portable FDSs are chiefly constrained by the hardware limitations (memory, computation capability, battery) of wearable devices.

Within the wearable category, inertial sensors, particularly accelerometers, gyroscopes, and in some cases magnetometers, represent the most widely used technology due to their compactness and low cost [10,11]. These sensors enable the characterization of kinematic patterns associated with falls, allowing them to be distinguished from conventional Activities of Daily Living (ADL) [12]. In this context, the performance of FDS is influenced not only by the sensor configuration or the body location of the device, but also by the algorithms used for signal analysis. Previous research has investigated different methodologies, including rule-based thresholds [8,13,14,15,16], machine learning (ML) techniques, and deep learning (DL) frameworks. Threshold-oriented methods are valued for their simplicity and low computational cost, whereas ML and DL models generally provide superior accuracy, though at the price of higher system complexity and greater energy consumption [12,16,17,18,19]. Recently, hybrid approaches have been proposed that combine a preliminary detection stage using threshold-based methods, which take advantage of their simplicity and low energy consumption. These approaches apply ML/DL techniques in a subsequent stage to refine classification and reduce false positives, achieving a balance between efficiency and performance [20,21,22].

A particularly critical aspect in this type of system is the sampling frequency of inertial sensors. The literature reveals a wide variability in the frequencies employed, ranging from 10 Hz to values as high as 800 Hz [23]. In this context, several studies have explored its impact on activity recognition.

As early as 2006, U. Maurer et al. [24] introduced the eWatch device, a multisensor wrist-worn platform equipped with a biaxial accelerometer designed to recognize various body postures. The authors evaluated several classification algorithms, including Decision Trees, k-Nearest Neighbors (k-NN), Naïve Bayes, and Bayes Net, and concluded that Decision Trees offered the most suitable trade-off between accuracy and computational requirements. The recognition accuracy improved as the sampling frequency increased, reaching stability between 15 and 20 Hz, which ultimately guided the adoption of a 20 Hz sampling rate in the final implementation.

Santoyo-Ramón et al. [25] carried out a systematic investigation on how sampling frequency influences wearable fall-detection systems based on accelerometer data. In their work, they implemented a Convolutional Neural Network (CNN) model, which was trained and evaluated using 15 publicly available datasets. Through progressive decimation of the time series, they observed that sensitivity and specificity remained above 95% for frequencies in the 15–20 Hz range. Noticeable degradation occurred only below 10–15 Hz. The power spectrum analysis and low-pass filtering experiments at 10–20 Hz corroborated the dominance of low-frequency components of human movements, leading the authors to recommend approximately 20 Hz as a compromise between performance and energy consumption. A similar conclusion has been reached in other studies [26,27,28,29] that have analyzed the relationship between the frequency content and the biomechanics a conventional human movement during daily life activities.

In contrast, Ajerla et al. [30] evaluated sampling frequency in a real-time wearable fall-detection system deployed at the edge. Using accelerometer data collected between 12.5 Hz and 200 Hz, and an LSTM-based classifier, they reported that a sampling rate of 50 Hz achieved the best detection performance, highlighting the influence of system-level factors beyond signal frequency content.

Additionally, Liu et al. [31] examined how varying sampling frequencies influence wearable fall-detection systems that utilize machine learning techniques. Their analysis combined data from the public SisFall repository and a proprietary dataset. By gradually decreasing the sampling frequency from 200/128 Hz down to 3 Hz, they assessed the performance of several classifiers, including Support Vector Machines (SVM), k-Nearest Neighbors (k-NN), Naïve Bayes (NB), and Decision Trees (DT). The findings indicated that a sampling frequency close to 22 Hz was sufficient to maintain an accuracy of at least 97% in most models, while the SVM with a radial basis kernel achieved comparable outcomes even at frequencies as low as 5.8 Hz. Concerning this, the choice of sampling frequency becomes particularly relevant in Internet of Things (IoT) scenarios, where wearable devices are frequently linked through energy-efficient wide-area communication systems, for instance LoRaWAN and Sigfox. In such systems, the available bandwidth and transmission time are strongly constrained [32]. Transmitting data at high frequencies increases energy consumption and reduces the device’s autonomy. By contrast, reducing the sampling rate decreases data volume, accelerates transmission, and extends battery life without compromising system accuracy [31]. Table 1 summarizes the studies presented above, highlighting the sampling frequencies, algorithms, and reported performance.

Despite the advances achieved in wearable fall-detection systems, several limitations persist in prior work. Many studies adopt fixed accelerometer sampling frequencies without systematic justification, often based on empirical choices, which prevents a clear assessment of their impact on detection performance, model stability, and computational efficiency [24,25,31]. In addition, most approaches focus on a single class of algorithms, either traditional machine learning or deep learning, limiting fair comparisons across modeling strategies and the evaluation of hybrid solutions under a uniform experimental protocol [33,34,35]. Furthermore, evaluations are frequently conducted using simulated falls under laboratory conditions, with limited validation on real-fall datasets or continuous real-life monitoring scenarios [36,37]. Finally, the practical implications of sampling frequency on energy consumption, data volume, and device autonomy, which are critical factors in IoT-based wearable systems, are often overlooked.

Therefore, this study aims to determine and validate the optimal sampling frequency that ensures the best balance between accuracy and sensitivity in wearable fall-detection models. Four sampling rates (10, 20, 50, and 100 Hz) were analyzed using the SisFall dataset [38], evaluating both deep learning architectures (CNN-LSTM, CNN, and LSTM with batch normalization) and classical machine learning algorithms (k-NN with 5 and 15 neighbors, and SVM). Beyond previous works, this study extends the analysis through a multi-stage validation, including assessments on external real-fall datasets (FARSEEING and Free From Falls) and on a custom wearable prototype operating at 20 Hz. The objective is to identify the sampling frequency that provides the most stable and accurate performance, confirming its suitability for fall detection under real-use conditions.

## 2. Methodology

### 2.1. Data and Preprocessing

The SisFall dataset [38], one of the most popular repositories considered by the fall-detection literature [29], was used in this study. This dataset comprises a total of 4505 motion recordings (2707 ADL and 1798 simulated falls) collected from 38 participants (19 older adults and 19 young subjects) [39]. Each record contains triaxial acceleration and angular velocity signals sampled at 200 Hz using sensors placed on the waist. The experimental protocol includes 15 types of falls (frontal, lateral, backward, from standing, while sitting, syncope, trips, and slips, among others) and 19 types of ADL (walking, climbing stairs, sitting, lying down, etc.), some of which exhibit motion patterns similar to falls. This similarity increases the classification challenge.

For the experimental setup, only the ADXL345 accelerometer (Analog Devices Inc., Wilmington, MA, USA) was used to record motion along the X, Y, and Z axes. Data fields corresponding to rotational parameters and measurements from a secondary accelerometer were omitted, as the proposed fall-detection method focuses exclusively on the acceleration magnitude derived from a single sensor.

The raw signals collected from the ADXL345 were converted into gravity units (g) to ensure accurate physical interpretation. The conversion followed the specifications of the sensor, taking into account its 13-bit resolution and a measurement range of ±16 g, as expressed in the following equation:

(1) $$a\left(g\right)=\frac{2\times Range}{2^{Resolution}}\times D_{bits}$$

where a(g) represents the acceleration in gravity units, *Range* is the accelerometer’s measurement range, *Resolution* denotes the bit depth of the sensor, and $D_{bits}$ corresponds to the raw digital output. In this way, each digital reading was transformed into acceleration values expressed in g, providing a consistent basis for subsequent signal analysis.

To analyze the effect of sampling frequency, the original 200 Hz signals were processed and re-sampled in Python 3.12.12 within a Google Colab environment. In particular, a linear-phase FIR filter was applied to each trace using the firwin function [40], which was used to adjust the cutoff frequency to the corresponding Nyquist frequency for each new sampling rate. Subsequently, a polyphase resampling was performed with resample_poly function [41] to obtain equivalent versions of the time series in the dataset at 100, 50, 20, and 10 Hz. This approach enabled explicit control of antialiasing and precisely preserved the relevant spectral content at each frequency.

**Figure 1:**
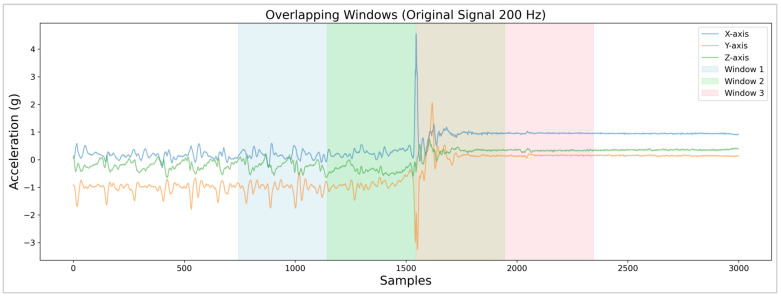
Four-second consecutive windows (with 50% overlap) extracted around the impact generated by a fall. A visible color change appears where two consecutive windows overlap.

Each record in the repository (representing a single action: fall or ADL) was segmented into 4 s windows centered on the maximum of the acceleration magnitude $\left(|\vec{a}|\right)$, which was computed from each instance of the acceleration components ($a_{x}$, $a_{y}$ and $a_{z}$) using Equation (2). In the case of the series generated during falls, this maximum corresponds to the instant of impact of the body against the floor. The selected 4 s interval is wide enough to represent the typical dynamics of both the pre-fall and post-fall phases [42]. In some cases, a 50% overlap was applied between consecutive windows to increase the number of available samples while controlling the redundancy between adjacent temporal segments, as illustrated in Figure 1, following a processing framework commonly adopted in wearable fall-detection studies [12].

(2) $$|\vec{a}|=\sqrt{a_{x}^{2}+a_{y}^{2}+a_{z}^{2}}$$

The resulting segments were exported as CSV (Comma Separated Values) files, merged into a single dataset, and cleaned to retain only the accelerometer magnitude, which was normalized using the Z-score technique, along with the corresponding binary classification label describing the movement (fall or ADL). The obtained patterns (a set of 4 s sequence of acceleration magnitude plus the labels) were then used to generate the training, validation and testing data. For model evaluation, a subject-independent protocol with ten participants was adopted. Six individuals were assigned to training, two to validation, and two to testing, ensuring that no participant was included in more than one subset. This experimental design provided a realistic estimation of model performance, consistent with the methodological approach described by Salah et al. [12].

### 2.2. Evaluated Models

Five representative approaches from the fall detection literature were evaluated, considering their diversity in computational complexity and modeling capability. All models were trained and validated using resampled signals at 10, 20, 50, and 100 Hz, allowing for a consistent comparative analysis. The configuration of each model was defined based on a combination of previous evidence from the literature [33,34,35,43,44,45,46] and exploratory experiments conducted in our environment [37,38,39,40,41,42,43].

For the advanced neural architectures, including CNN, LSTM combined with batch normalization, and CNN–LSTM models, a grid search was initially applied to explore hyperparameters, evaluating learning rates in the range of 10^−5^ to 10^−1^ and convolutional filter sizes between 16 and 256, followed by empirical fine-tuning using validation accuracy as the main criterion. The number of epochs and batch size were kept constant to ensure comparability. For k-NN, values of K = 1–20 were tested on the validation set, selecting K = 5 and K = 15 as representative configurations. For SVM, a linear kernel with C = 1.0 was chosen, consistent with related studies and representing a balance between bias and variance. Each training process was repeated five times to verify result stability, and the best-performing model from each configuration was selected for further analysis. Table 2 summarizes the final configuration of the models evaluated at the different sampling frequencies.

As shown in Table 2, the configurations illustrate a balance between more complex architectures, capable of capturing temporal and spatial dependencies (CNN, LSTM + BN, and CNN-LSTM), and classical models with lower computational cost (k-NN and SVM). This diversity allowed the evaluation not only of performance in terms of accuracy, but also of the practical implications related to model complexity and training time, aspects that are analyzed in the following sections.

### 2.3. Experimental Settings

All experiments were conducted using Python 3.12.12 within the Google Colab cloud-computing environment [47]. Signal filtering and resampling were performed using SciPy v1.16.3, classical machine learning models were implemented with Scikit-learn v1.6.1, and deep learning architectures were developed using TensorFlow/Keras v2.19.0. Model training and evaluation were accelerated using an NVIDIA Tesla T4 GPU, as provided by the Colab runtime, ensuring efficient execution and reproducibility of the experiments.

### 2.4. Evaluation Metrics

To evaluate the effectiveness of the models in detecting falls, we compute five standard indicators commonly considered to assess binary classifiers: accuracy, sensitivity (or recall), specificity, precision (positive predictive value), and F1-score. These indicators were obtained from the confusion matrix and describe both the proportion of falls correctly recognized and the model’s capability to differentiate them from activities of daily living (ADL) [34]. They are formally defined as follows:

(3) $$\mathrm{Accuracy}=\frac{TP+TN}{TP+TN+FP+FN}$$

(4) $$\mathrm{Sensitivity}=\frac{TP}{TP+FN}$$

(5) $$\mathrm{Specificity}=\frac{TN}{TN+FP}$$

(6) $$\mathrm{Precision}=\frac{TP}{TP+FP}$$

(7) $$F1\text{-}\mathrm{score}=2\times \frac{\mathrm{Precision}\times \mathrm{Sensitivity}}{\mathrm{Precision}+\mathrm{Sensitivity}}$$

In these equations, *TP* (True Positives) refers to the number of fall events correctly recognized by the model, whereas *TN* (True Negatives) corresponds to activities of daily living (ADLs) that were accurately identified as non-fall events. Similarly, *FP* (False Positives) indicates instances of ADL that were mistakenly classified as falls, and *FN* (False Negatives) represents real falls that went undetected. Sensitivity is directly linked to the proportion of actual falls that the models are able to identify, while Specificity describes the ability of the classifiers to avoid false alarms. Precision in turns quantifies how many of the detected events were actual falls, while Accuracy represents the global hit ratio of the models and the F1-score reflects the harmonic mean between precision and sensitivity, serving as a more balanced metric of overall detection performance. For all neural models, a fixed probabilistic decision threshold of 0.5 was applied to the sigmoid output to convert predicted probabilities into binary class labels (fall/no-fall) when computing these metrics.

### 2.5. Experimental Validation of the Selected Model

**Figure 2:**
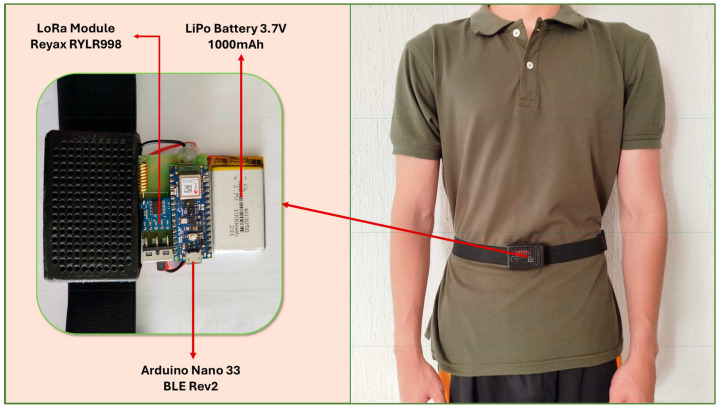
Hardware setup and on-body placement of the wearable device [11].

Based on the comparative results, the model that demonstrates the best overall performance will undergo an additional validation process. This stage will be conducted at the sampling frequency identified as optimal in our experiments and will comprise two complementary steps: (i) the evaluation of the system using external datasets of real-world falls, specifically FARSEEING [36] and the Free From Falls (FFF) study [48], in order to verify the model’s generalization capability; and (ii) implementing and testing the selected classifier on a wearable device based on the Arduino Nano 33 BLE Sense Rev2 (Arduino S.r.l., Monza, Italy) [49], which incorporates a triaxial accelerometer positioned at the waist, as shown in Figure 2, to assess its performance under conditions closer to real-world use (see our work in [11] for further details of the employed prototype of FDS).

## 3. Results

### 3.1. Comparative Performance Across Sampling Rates

To analyze the impact of temporal resolution on fall detection, a comparative evaluation was conducted using five representative model types—CNN, LSTM (Long Short-Term Memory) with batch normalization, a combination of CNN and LSTM, k-NN (evaluated with k = 5 and k = 15), and SVM—at four sampling frequencies (10, 20, 50, and 100 Hz). Table 3 presents the results in terms of accuracy, sensitivity, and specificity. For learning-based models, the reported Best Accuracy (%) corresponds to the best-performing run among five independent executions, while Accuracy (mean (%) ± σ) reflects the average performance and variability across these runs; sensitivity and specificity are reported for the best-performing run. As can be observed from the table, deep learning frameworks consistently achieved superior performance compared with traditional machine-learning models, emphasizing the strong impact of the sampling frequency on the effectiveness of several models in differentiating falls from activities of daily living (ADL).

**Figure 3:**
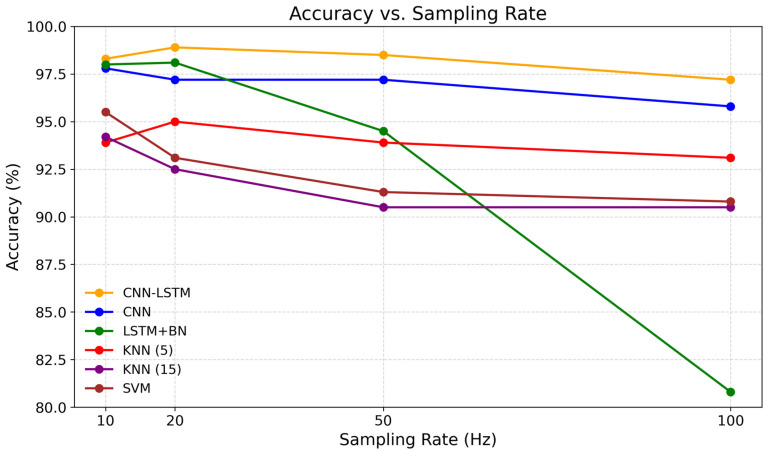
Accuracy vs. Sampling Rate.

As illustrated in Figure 3, the CNN-LSTM model achieved the highest and most stable performance, peaking at 20 Hz (accuracy = 98.89%, specificity = 99.63%). This result confirms that intermediate sampling rates effectively capture the distinctive motion dynamics of falls while minimizing redundancy and low-frequency noise often associated with ADL. In contrast, the standalone LSTM model showed a pronounced drop in accuracy above 50 Hz, suggesting that excessive temporal resolution may interfere with the preservation of sequential dependencies while unnecessarily increasing model complexity and the number of trainable parameters. Meanwhile, classical approaches such as KNN and SVM exhibited limited adaptability, with noticeable degradation at 100 Hz.

#### Controlled Evaluation Using a Unified CNN-LSTM Architecture

To isolate the effect of sampling frequency from architectural variability, an additional controlled experiment was conducted using a unified CNN-LSTM architecture across all sampling rates. For 10 Hz and 20 Hz, the CNN-LSTM architecture was already identical, whereas for 50 Hz and 100 Hz the same configuration was enforced to eliminate architectural tuning effects. The performance obtained with this unified architecture is summarized in Table 4. As can be observed, the performance trends reported in Table 3 are preserved, confirming that the observed differences are mainly driven by sampling frequency rather than architectural adjustments.

### 3.2. Optimal Model Configuration and In-Depth Evaluation

After comparing all models across sampling rates, the optimal configuration for each algorithm was determined to facilitate performance comparison. Table 5 summarizes these best results per model, highlighting the sampling rate at which each achieved its highest accuracy, sensitivity, and specificity.

As shown, deep learning models maintained superior performance across all sampling rates, with the CNN-LSTM at 20 Hz achieving the most balanced results (accuracy = 98.89%, sensitivity = 96.67%, specificity = 99.63%). The LSTM + Batch Normalization model followed closely, confirming that architectures capable of modeling temporal dependencies outperform traditional classifiers such as SVM and k-NN, which exhibit noticeable degradation in sensitivity. These findings are consistent with previous studies, where hybrid and recurrent models demonstrated improved robustness and generalization in fall-detection tasks [44,45].

The predominance of intermediate frequencies (10–20 Hz) among the best configurations supports prior evidence that most discriminative motion dynamics related to falls occur below 20 Hz, where signal information remains sufficiently rich while computational and energy costs are reduced [25]. Consequently, the CNN-LSTM at 20 Hz was selected for an in-depth evaluation of its learning behavior, convergence stability, and classification reliability.

**Figure 4:**
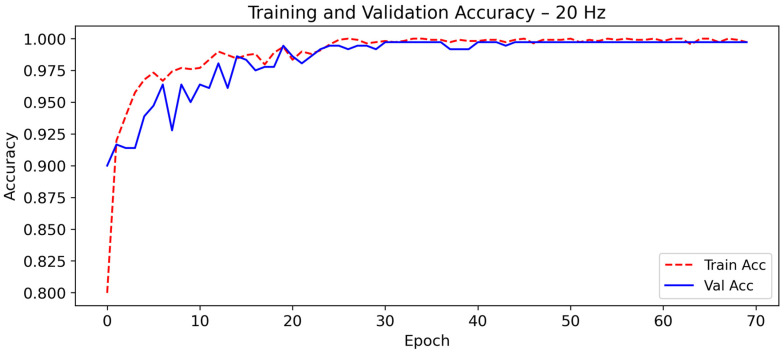
Evolution of the training and validation accuracy of CNN-LSTM model at 20 Hz as a function of the number of epochs during training.

**Figure 5:**
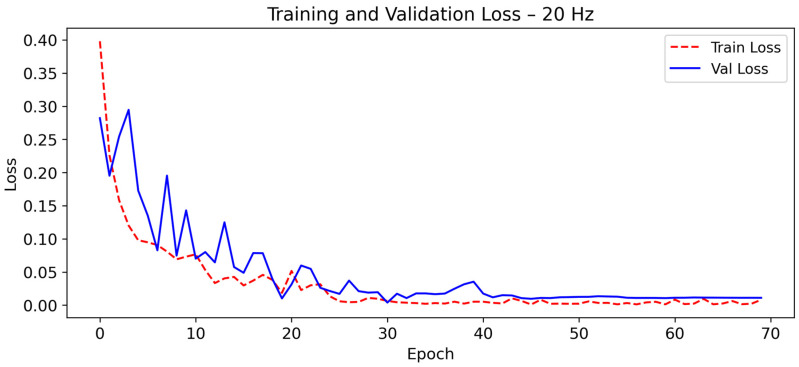
Evolution of the training and validation loss of CNN-LSTM model at 20 Hz as a function of the number of epochs during training.

As shown in Figure 4 and Figure 5, the model exhibits rapid convergence during the first 15 epochs. After this point, both training and validation curves stabilize with minimal fluctuations.

The close alignment between training and validation curves confirms the strong generalization capability of the model and the absence of overfitting. This stability is typical of well-regularized CNN-LSTM architectures, where convolutional layers extract discriminative motion features and LSTM units capture temporal dependencies necessary to distinguish falls from ADL. Similar robustness has been reported in multimodal activity-recognition networks [50,51], confirming the suitability of this hybrid topology for noise-tolerant and energy-efficient fall detection [52].

**Figure 6:**
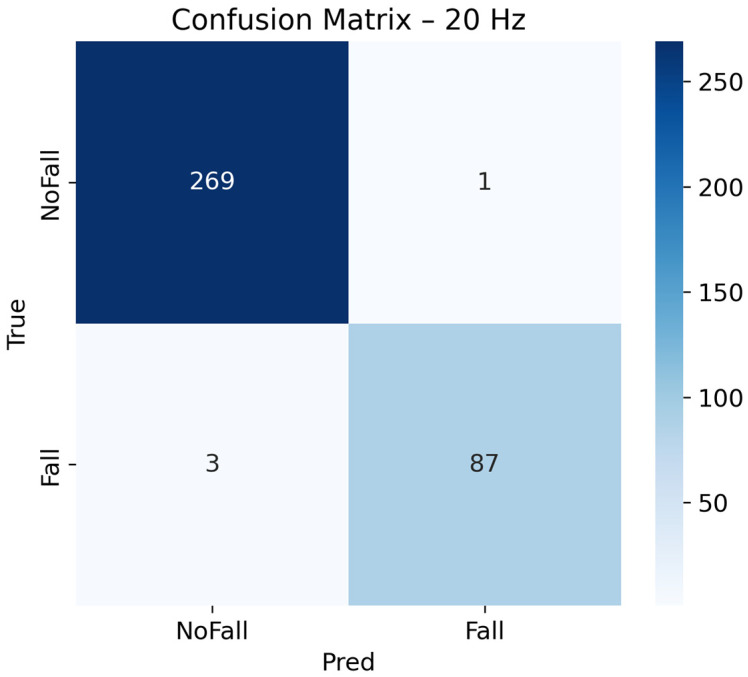
Confusion matrix of CNN-LSTM at 20 Hz on the test set.

The confusion matrix in Figure 6 further corroborates these findings. Out of 270 non-fall activities in the test set, 269 were correctly classified, and out of 90 fall events, 87 were correctly identified. Thus, the classifier only generated one false positive and three false negatives.

These results yield a precision (PPV) and negative predictive value of ≈98.9%, with an F1-score of 97.8%, confirming the strong discriminative capability of the CNN-LSTM model. The low false-alarm rate is particularly relevant in remote-monitoring scenarios, where excessive false positives can cause caregiver alert fatigue [53]. Overall, the empirical optimum at ≈20 Hz aligns with previous studies and represents the best trade-off between accuracy and energy efficiency for IoT-based wearable fall-detection systems [25,54,55].

### 3.3. Validation on External Datasets (FARSEEING and FFF)

To assess the generalization capability of the model beyond the training environment, an additional cross-dataset evaluation was conducted using two external repositories containing real fall events: FARSEEING [36] and FFF (Free From Falls) project [48] datasets. In this phase, the optimal configuration of the CNN-LSTM model, previously trained and validated on the SisFall dataset, was applied at a sampling frequency of 20 Hz, which had yielded the most balanced performance across all tested rates. For this purpose, the original series in these two datasets were resampled to the common frequency of 20 Hz following the procedure described in Section 2.1. The experiments were executed in Google Colab (v2024.10) [47], automating the segmentation of windows, inference, and computation of performance metrics. Both datasets include accelerometer signals recorded under real-life conditions but differ in population characteristics and acquisition protocols, allowing the evaluation of the model’s robustness against variations in environment and sensor placement.

**Figure 7:**
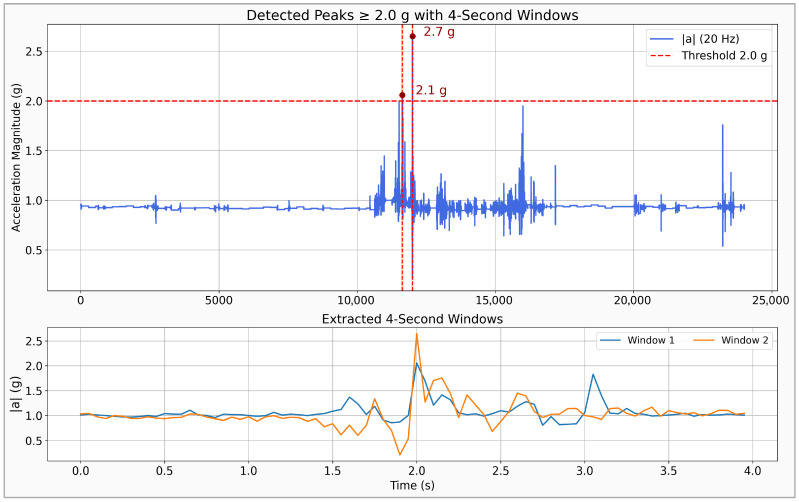
Examples of the detected maxima of the acceleration module and 4 s windows extracted from one trace in FARSEEING dataset. The dotted horizontal line indicates the acceleration threshold, while the dotted vertical lines mark acceleration peaks that exceeded this threshold.

The FARSEEING dataset [36], developed by the European consortium of the same name, comprises more than 200 real falls recorded between 2012 and 2015 in older adults during daily life activities. The sensors, one triaxial accelerometer positioned on the subject’s lower back or thigh, operated at sampling rates of 20 Hz or 100 Hz. Figure 7 illustrates one of the movements from the FARSEEING dataset, highlighting the detected peaks and the corresponding 4 s windows that were extracted based on those peaks to test the model. Following this procedure on 22 extended recordings, 104 four-second windows with accelerations equal to or above 2 g were extracted. Due to missing timestamps, the absolute maximum peak per recording was labeled as the fall, whereas secondary peaks were treated as ADLs. These samples were analyzed using the CNN-LSTM model with a probabilistic threshold of *p* > 0.4. The model correctly detected 18 of the 22 real falls (sensitivity = 81.8%), produced three false detections, and correctly classified 79 of 82 non-fall windows, achieving an overall accuracy of 93.3% and a specificity of 96.3%. The false alarms corresponded to abrupt but non-fall movements, whereas the missed events involved lower-amplitude acceleration patterns.

The Free From Falls (FFF) project dataset [48] contains the traces captured during real fall episodes from individuals with multiple sclerosis, recorded during eight weeks of continuous monitoring in their homes using a triaxial accelerometer with a sampling rate of 50 Hz. The sensors were uniformly placed on the subjects’ lower back, ensuring consistent signal acquisition across participants. To process the continuous stream, a pre-selection threshold of 1.1 g was applied to extract candidate four-second windows. Additionally, a minimum separation of 40 s was enforced between detections to prevent counting the same fall event multiple times. Using a classification threshold of 0.85 for the CNN-LSTM model, 690 four-second windows extracted from the 49 FFF traces were analyzed. The test with the trained model yielded 48 true positives, one false negative, and seven false positives, resulting in an accuracy of 98.3%, a sensitivity of 97.9%, and a specificity of 98.9%, confirming its high reliability under controlled experimental conditions and traces captured during real falls.

Compared to FARSEEING (accuracy = 93.3%, sensitivity = 81.8%), this superior performance is mainly attributed to the greater homogeneity in sensor placement and recording conditions in FFF, while the variability in sensor locations and sampling rates in FARSEEING may have introduce kinematic diversity and a slightly reduced sensitivity.

### 3.4. Experimental Validation on the Wearable Device

Following the evaluation with external datasets containing real fall events, an additional validation was conducted to examine the system’s stability and the occurrence of false alarms under real-world usage conditions. To this end, the model was implemented on the wearable prototype based on the Arduino Nano 33 BLE Sense Rev2 presented in [11]. The device incorporated a triaxial accelerometer positioned at the waist and was configured to sample data at 20 Hz with an threshold of 2 g for the measured acceleration module. Each time this threshold was exceeded, the system automatically generated a 4 s window, which was subsequently processed by the CNN-LSTM model using a probabilistic decision threshold of 0.5.

The real-life validation was performed over seven consecutive days, during which the participant transporting the detector engaged in routine low-intensity daily activities such as walking, brief runs to cross streets, climbing stairs, riding a motorcycle, traveling as a taxi passenger, working in an office environment, eating, and performing common household tasks. During this period, the device analyzed 1147 observation windows that were suspected of being caused by a fall after exceeding the acceleration threshold. Of these, 58 were incorrectly classified as falls, yielding a false-positive rate of 5.06% and a specificity of 94.94%.

Although no actual falls occurred during the trial, the results demonstrate the model’s ability to maintain stable classification performance when exposed to typical daily life movements and its low tendency to trigger false alarms, confirming its reliability in real-world continuous-monitoring scenarios.

**Figure 8:**
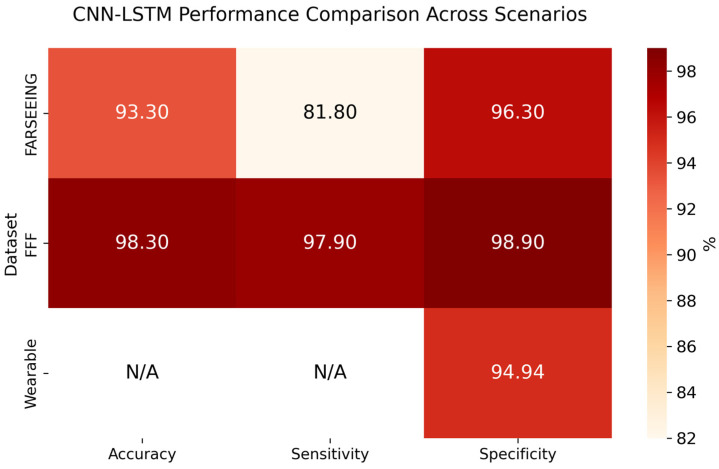
CNN-LSTM performance across external validation and real-life data from the wearable.

As shown in Figure 8, the CNN-LSTM model preserved stable and consistent performance across all validation scenarios. The comparison among FARSEEING, FFF, and wearable evaluations highlights its robustness to variations in data acquisition and its reliability under continuous monitoring conditions.

## 4. Discussion

The obtained results show that the sampling frequency may exert a noteworthy influence on the performance of wearable Fall Detection Systems (FDS), revealing an optimal point around 20 Hz, where the highest detection capability is achieved without generating unnecessary data volume. Consistent with previous studies, we found that sampling rates above approximately 50–100 Hz yield marginal or negligible improvements in accuracy. Moreover, in some deep learning–based models, increasing the sampling rate can even lead to a degradation in the quality metrics of the detection decision. Santoyo-Ramón et al. [25] reported that a frequency of 15–20 Hz is sufficient to maintain sensitivities and specificities above 95%, while Liu et al. [31] found that 22 Hz was enough to achieve ~97% accuracy across multiple classifiers. Our experiments fully support these conclusions: increasing temporal resolution beyond ~20 Hz did not enhance model performance and, in the case of the pure LSTM architecture, even caused a sharp drop in accuracy at 100 Hz. This phenomenon suggests that, beyond a certain threshold, the inclusion of additional samples may hinder the correct modeling of temporal dependencies without providing relevant kinematic information. In fact, higher sampling frequencies reduce the parsimony of the system by increasing the dimensionality of the input space, which most likely impacts the model’s ability to extract discriminative features. Thus, higher-rate data may introduce redundancy that causes overfitting, which may ultimately challenge the network’s generalization capacity. In contrast, moderate reductions in sampling rate did not compromise fall detection, indicating that the distinctive dynamics of these events are predominantly concentrated in low frequencies. In fact, several models exhibited stable performance even at 10 Hz, consistent with the findings of Maurer et al. [24], who observed that movement recognition hit rate in a HAR system using with an eWatch improved up to ~15–20 Hz before reaching an accuracy plateau above 20 Hz. Although our CNN achieved its best sensitivity at 10 Hz (slightly higher than at 20 Hz), the difference was minimal, suggesting that even 10 Hz may be viable in certain scenarios. Nevertheless, selecting ~20 Hz provides a safety margin to capture the rapid transients of falls without incurring a significant computational cost, aligning with the trade-off between performance and energy efficiency reported in the literature [25,31,55].

A second critical aspect concerns the performance gap between deep learning architectures and traditional machine learning methods, as well as their practical implications. In our experiments, the CNN-LSTM architecture clearly outperformed SVM and k-NN at 20 Hz (98.89% accuracy versus 93.06% and 95.00%, respectively), reflecting its superior ability to capture the nonlinear and temporal patterns characteristic of falls. This behavior has also been reported in previous studies, such as that of Afuan et al. [56]. These deep-learning approaches typically require balanced datasets and extensive preprocessing. Given these computational demands, a hybrid pipeline was implemented wherein the wearable locally stores 4 s windows upon detecting peaks > 2 g, which are subsequently analyzed externally using the CNN-LSTM model. Operating at 20 Hz, this configuration yielded a false-positive rate below 1.5% in controlled tests and a cumulative error of 5% during seven-day continuous monitoring. This equates to approximately 8 false alarms per day, a rate consistent with most clinical trials [37]. It is worth noting that most false positives originated from abrupt but non-fall activities such as climbing stairs quickly, riding a motorcycle, running, or traveling by taxi, yet the model filtered out the vast majority of these events.

From a practical standpoint, our results indicate that it is feasible to design wearable fall detection systems that are both highly accurate and energy-efficient by limiting the sampling frequency to approximately 20 Hz. To quantify this efficiency, our empirical measurements using an ERASMUS EMT-500 multimeter revealed a ~44% reduction in sensor current (2.616 mA at 100 Hz vs. 1.459 mA at 20 Hz). Furthermore, the trained model size dropped by ~62% (2705 KB to 1030 KB) and the transmission payload by 80% (15 KB to 3 KB). These combined factors directly translate to a lower computational and transmission load, resulting in a significant extension of the device’s battery life [25,57]. At the same time, the false alarm rate remained low (specificity ~95% during daily life testing), which is a critical factor for system acceptance since excessive false alarms can lead to desensitization among caregivers and users [53,58]. In our external validations, the CNN-LSTM model trained with the SisFall dataset demonstrated remarkable generalization capability, achieving about 82% sensitivity in detecting real falls from the FARSEEING repository of acceleration signals captured during actual falls (with ~96% specificity). This performance is consistent with that reported by Bourke et al. [58], who, using a machine learning algorithm, obtained 88% sensitivity and 87% specificity with real data from the same dataset. Similarly, in the FFF repository, which features more homogeneous conditions, the model achieved nearly 98% sensitivity, confirming its robustness when the sensor configuration remains consistent. Overall, this study provides a comprehensive evaluation that unifies and extends previous findings, offering quantitative guidelines to optimize the sampling rate without compromising effectiveness. This is particularly valuable for the development of wearable IoT devices, as it enables more durable and reliable telemonitoring solutions capable of improving the safety and quality of life of vulnerable populations.

## 5. Conclusions

This study provides clear experimental evidence that the sampling frequency strongly shapes the performance and stability of wearable fall-detection models. The obtained results show that a CNN-LSTM architecture trained at 20 Hz achieved the most consistent balance between sensitivity and specificity across synthetic and real-world datasets, including data captured during a seven-day continuous monitoring campaign with a functional prototype. These findings highlight that moderate sampling frequencies can effectively capture the kinematic signatures of falls while maintaining model reliability under everyday conditions. Overall, the results establish a practical reference for developing next-generation wearable systems for fall detection, combining accuracy, robustness, and long-term operational feasibility within IoT-based monitoring environments.

## Figures and Tables

**Table 1:** Summary of studies on sampling frequency in wearable fall-detection systems.

Model	Algorithm/ Model	Sampling Frequency (Hz)	Main Findings
Maurer et al. [24]	DT, k-NN, NB, Bayes Net	1–30 (downsampled from 50 Hz)	Accuracy stabilized at 15–20 Hz; Decision Trees showed the best accuracy–complexity trade-off; final system implemented at 20 Hz
Santoyo-Ramón et al. [25]	CNN	1–140 Hz (effective, via decimation)	Sensitivity and specificity > 95% at 15–20 Hz
Liu et al. [31]	SVM (RBF), k-NN, NB, DT	3–200 (200/128 Hz original)	Accuracy ≥ 97% at ~22 Hz; SVM remains effective at lower sampling rates.
Ajerla et al. [30]	LSTM (edge framework)	12.5, 25, 50, 100 and 200 (real-time data collection)	50 Hz performed best; below 50 Hz missed falls; >50 Hz similar. Best overall system: 95.8% accuracy (waist + wrist).
This work	CNN-LSTM, CNN, LSTM, SVM, k-NN	10, 20, 50 and 100 (downsampled from 200 Hz)	20 Hz provides best accuracy–efficiency trade-off

**Table 2:** Model configurations across sampling frequencies after hyperparameter grid search.

Model	Common Parameters	Sampling Rate (Hz)	Model Configuration
CNN-LSTM	Window = 4 s; 70 epochs; 5 runs; Batch = 32; LSTM = 64–64; Dense = 128 + Dropout = 0.4; Optimizer = Adam; Loss = BCE; Output = Binary classification (Fall/No-Fall)	10	Conv(32,64), Dropout = 0.2/0.2, no PrePool
		20	Conv(32,64), Dropout = 0.2/0.2, no PrePool
		50	Conv(64,128), Dropout = 0.25/0.25, with PrePool
		100	Conv(128,256), Dropout = 0.3/0.3, with PrePool
CNN	Window = 4 s; 70 epochs; 5 runs; Batch = 32; 3 Conv + BN + Dropout + GAP; Dense = 128 + Dropout = 0.4; Optimizer = Adam; Loss = BCE; Output = Binary classification (Fall/No-Fall)	10	Conv(32,64), Dropout = 0.2, no PrePool
		20	Conv(32,64), Dropout = 0.2, no PrePool
		50	Conv(64,128), Dropout = 0.25, with PrePool
		100	Conv(128,256), Dropout = 0.3, with PrePool
LSTM + BN	Window = 4 s; 70 epochs; 5 runs; Batch = 32; LSTM(32,32) + BN + Dropout = 0.2; Dense = 64 + Dropout = 0.5; Optimizer = Adam; Loss = BCE; Output = Binary classification (Fall/No-Fall)	10	Window = 40 samples
		20	Window = 80 samples
		50	Window = 200 samples
		100	Window = 400 samples
k-NN	Window = 4 s; StandardScaler; Validation sweep K = 1–20; Test with fixed K (5 or 15); Metric = Accuracy; Output = Binary classification (Fall/No-Fall)	10	Window = 40 samples, K = 5 or 15
		20	Window = 80 samples, K = 5 or 15
		50	Window = 200 samples, K = 5 or 15
		100	Window = 400 samples, K = 5 or 15
SVM	Window = 4 s; Linear kernel; C = 1.0; StandardScaler; Optional kernels = RBF/Poly; Metric = ROC-AUC; Output = Binary classification (Fall/No-Fall)	10	Window = 40 samples
		20	Window = 80 samples
		50	Window = 200 samples
		100	Window = 400 samples

**Table 3:** Performance of All Models on SisFall at Different Sampling Rates.

Model	Sampling Rate (Hz)	Best Accuracy (%)	Sensitivity (%)	Specificity (%)	Accuracy (Mean (%) ± σ)
CNN-LSTM	10	98.33	96.67	98.89	97.56 ± 0.77
	**20**	**98.89**	**96.67**	**99.63**	**98.11 ± 0.54**
	50	98.61	97.78	98.89	97.33 ± 0.80
	100	97.22	97.78	97.04	96.06 ± 0.73
CNN	**10**	**97.78**	**96.67**	**98.15**	**97.61 ± 0.22**
	20	97.22	94.44	98.15	96.50 ± 0.52
	50	97.22	92.22	98.89	95.61 ± 1.13
	100	95.83	88.89	98.15	95.50 ± 0.27
LSTM (with Batch Norm)	10	98.06	93.33	99.63	96.72 ± 0.75
	**20**	**98.06**	**95.56**	**98.89**	**97.06 ± 0.89**
	50	94.44	96.67	93.70	89.72 ± 5.67
	100	80.83	37.78	95.19	79.94 ± 0.59
K-NN (5 neighbors)	10	93.89	83.33	97.41	N/A
	**20**	**95.00**	**83.33**	**98.89**	
	50	93.89	80.00	98.52	
	100	93.06	78.89	97.78	
K-NN (15 neighbors)	**10**	**94.17**	**83.33**	**97.78**	N/A
	20	92.50	70.00	100.00	
	50	90.56	64.44	99.26	
	100	90.56	62.22	100.00	
SVM	**10**	**95.56**	**93.33**	**96.30**	N/A
	20	93.06	90.00	94.07	
	50	91.39	90.00	91.85	
	100	90.83	90.00	91.11	

Bold values indicate the best performance for each model across the evaluated sampling rates. N/A denotes metrics not available for certain models.

**Table 4:** Performance of the CNN-LSTM model using a unified architecture across sampling rates.

Model	Common Parameters and Model Configuration	Sampling Rate (Hz)	Best Accuracy (%)	Sensitivity (%)	Specificity (%)
CNN-LSTM	Window = 4 s; 70 epochs; 5 runs; Batch = 32; LSTM = 64–64; Dense = 128 + Dropout = 0.4; Optimizer = Adam; Loss = BCE; Output = Binary classification (Fall/No-Fall); Conv(32,64), Dropout = 0.2/0.2, no PrePool	10	98.33	96.67	98.89
		20	98.89	96.67	99.63
		50	97.50	97.78	97.41
		100	97.22	98.89	96.67

**Table 5:** Best Results per Model on SisFall.

Model	Sampling Rate (Hz)	Best Accuracy (%)	Sensitivity (%)	Specificity (%)
CNN-LSTM	20 Hz	98.89	96.67	99.63
LSTM + BatchNorm	20 Hz	98.06	95.56	98.89
CNN	10 Hz	97.78	96.67	98.15
SVM	10 Hz	95.56	93.33	96.30
KNN (5 neighbors)	20 Hz	95.00	83.33	98.89
KNN (15 neighbors)	10 Hz	94.17	83.33	97.78

## Data Availability

The data presented in this study are openly available in Zenodo at https://doi.org/10.5281/zenodo.17776345.
